# *Saccharomyces boulardii* combined with triple therapy alter the microbiota in the eradication of *Helicobacter pylori* infection

**DOI:** 10.1038/s41598-024-63894-z

**Published:** 2024-06-07

**Authors:** Yan Zhang, Bingyun Lu, Yu Dong, Yifeng Zhang, Qianming Du, Ye Chen, Zhenyu Zhang

**Affiliations:** 1 Department of Gastroenterology, Nanjing First Hospital, Nanjing Medical University, 68 Changle Road, Nanjing, 210006 People’s Republic of China; 2 Department of Gastroenterology, Shenzhen Hospital, Southern Medical University, Shenzhen, Guangdong People’s Republic of China; 3 Integrative Microecology Clinical Center, Shenzhen Key Laboratory of Gastrointestinal Microbiota and Disease, Shenzhen Clinical Research Center for Digestive Disease, Shenzhen Technology Research Center of Gut Microbiota Transplantation, Shenzhen Hospital, Southern Medical University, 1333 Xinhu Road, Bao’an District, Shenzhen, 518101 People’s Republic of China

**Keywords:** *Saccharomyces boulardii*, *Helicobacter pylori* eradication, Intestinal flora, Microbiology, Gastroenterology

## Abstract

To assess the effectiveness and safety of combining *Saccharomyces boulardii* powder with triple therapy as a primary approach for eradicating *H. pylori* infection, a total of 144 patients who tested positive for *H. pylori* and diagnosed with non-ulcer dyspepsia underwent endoscopy at two national centers between June 2017 and March 2019 were included. The patients were categorized into three groups using a subsection randomization method and received initial *H. pylori* eradication treatments. Microbial composition, eradication rates, symptom alleviation, and adverse reactions were monitored on the 14th and 44th days post-treatment. According to PP analysis showed the eradication rates for the SRAC group was 75%, BRAC was 93.18% and RAC was 65.2%. Group BRAC exhibited a marginally higher eradication rate compared to other groups. However, patients receiving *Saccharomyces boulardii* treatment exhibited an overall reduction in initial dyspepsia symptoms by the end of the treatment period. When employed as a primary strategy, the combination of *Saccharomyces boulardii* powder with triple therapy displayed notable efficacy and smaller gastrointestinal side effects in eradicating initial *H. pylori* infections among non-ulcer dyspepsia patients. Moreover, this approach demonstrated advantages in alleviating symptoms, exhibited favorable tolerance, and maintained a high level of clinical safety.

## Introduction

*Helicobacter pylori* (HP) stands as one of the most prevalent chronic infections in humans, bearing a close association with various upper gastrointestinal and extragastrointestinal disorders^1^. Comprehensive epidemiological studies in China underscore the potential of HP eradication in preventing gastric cancer and reversing precancerous lesions like gastric mucosal atrophy and intestinal metaplasia^2^. In 1994, the World Health Organization/International Agency for Cancer Research (WHO/IARC) designated *Helicobacter pylori* as a Class I carcinogen. The latest meta-analysis and epidemiological survey show that in recent years, the personal infection rate of *Helicobacter pylori* in Chinese Mainland is about 44.2%, but the domestic infection rate in China is as high as 71.21%^3,4^. *Helicobacter pylori* is closely related to gastric cancer, so eradication is vital importance. The expanding landscape of HP treatment has brought the challenge of drug resistance to the forefront, compromising the efficacy of eradication efforts. Presently, China recommends a 14-day, four-drug regimen featuring two antibiotics as the primary treatment, aimed at elongating the treatment duration and intensifying drug combinations to bolster eradication rates. However, this approach correlates with elevated adverse reactions^5^. In response, incorporating micro-ecological agents, mucosal protectors, and traditional Chinese medicine has emerged as a novel strategy to combat drug resistance and optimize eradication efficacy. Notably, the concept of “fighting bacteria with bacteria” has heightened interest in the role of probiotics in eradication treatment^6^.

Upon revisiting the role of probiotics in *Helicobacter pylori* eradication, in vitro experiments demonstrate the potential of lactobacillus (*Lactobacillus acidophilus*) or its byproducts to inhibit or eliminate *Helicobacter pylori*. Animal studies (involving *Lactobacillus acidophilus*) and clinical trials corroborate the efficacy of probiotics (including bifidobacterium, *Lactobacillus acidophilus*, and *Enterococcus* triple live bacteria) in bolstering the success of PPI triple therapy for *Helicobacter pylori* eradication. These probiotics also exhibit a capability to mitigate overall adverse reactions and specific symptoms, such as diarrhea, upper abdominal pain, nausea, and taste disruptions^7,8^. Nevertheless, the effectiveness of individual probiotic preparations varies, emphasizing the need for a targeted evaluation of single probiotics against *Helicobacter pylori* infection.

Among probiotic preparations, *Saccharomyces boulardii* (*S.* *boulardii*) reigns as a widely utilized contender. Homeyoung Zojaji et al. randomly enrolled 160 adult patients with biopsy confirmed *Helicobacter pylori* infection and divided them into a standard triple therapy combined with *Saccharomyces boulardii* group and a standard triple therapy group. The results showed no significant difference in eradication rates between the experimental group and the control group (87.5% vs. 81.2%)^9^. Similar results also appeared in another clinical study targeting children (71.4% vs. 61.9%, *p* > 0.05). The research results suggest that *Saccharomyces boulardii* can improve the eradication rate of *Helicobacter pylori* and reduce treatment-related side effects, especially diarrhea^10^. Nevertheless, another randomized controlled study compared standard triple therapy, *Saccharomyces boulardii* + standard triple therapy, and sequential therapy (89.1% vs. 77.1% vs. 95.5%, *p* < 0.05), and the results indicated that probiotic therapy was not superior to sequential therapy^11^. Research highlights the potential of *Saccharomyces boulardii* in enhancing eradication efficacy and reducing adverse reactions during *Helicobacter pylori* eradication. Nonetheless, the combined use of *Saccharomyces boulardii* with standard PPI triple therapy remains a subject of debate concerning both efficacy and safety. Moreover, the impact of this treatment on intestinal micro-ecology remains inadequately explored^9,10^. Therefore, this study aims to assess the effectiveness and safety of *Saccharomyces boulardii* powder when combined with triple therapy for *Helicobacter pylori* eradication. It also seeks to elucidate the influence of *Helicobacter pylori* treatment on intestinal micro-ecology and the predictive role of intestinal flora in treatment outcomes.

## Material and methods

### Patient enrollment

This clinical trial has been registered with the China Clinical Trial Registration Center (ChiCTR-IOR-16008610, 07/06/2016) and was approved by the Ethical Committee of Nanjing First Hospital, Nanjing Medical University (KY20160721-01). All methods were performed in accordance with the relevant guidelines and regulations. Between June 2017 and March 2019 from 2 medical centers in China, 144 patients between the ages of 18 and 65 years were enrolled in this study after receiving endoscopic evaluations for various GI symptoms. Each of these patients had rapid urease test proof of *H. pylori* infection, diagnosed as NUD and underwent initial HP eradication treatment. The inclusion criteria included (1) The diagnosis must be obtained through gastroscopy (within 1 month); (2) Positive for rapid urease test, positive for urea breath test within 2 weeks before or 1 month after gastroscopy; (3) Age 18 ~ 65 years old, gender unlimited; (4) Has not received formal eradication treatment of *Helicobacter pylori* in the past; (5) Have not taken any probiotics recently; (6) Sign informed consent. The exclusion criteria included (1) Those who have used antibiotics, bismuth agents or H2 receptor antagonist (H_2_RA) or proton pump inhibitor (PPI) within 4 weeks before treatment; (2) Pregnant or lactating women; (3) The patient also has other serious diseases that affect the evaluation of this study, such as severe liver disease, heart disease, cerebrovascular disease, kidney disease, malignant tumor and alcoholism; (4) Those who are allergic to the drugs used in this study; (5) Those who have participated in other drug research within 3 months; (6) Patient unable to correctly express their symptoms and wishes, unable to cooperate with the experimenter. Termination test criteria (1) Those who have serious adverse reactions during the test and cannot tolerate them; (2) Other diseases that interfere with the test during treatment; (3) Lost visit; (4) Pregnancy during treatment. We obtained informed consent from all subjects and their legal guardians.

### Study design

This was a randomized, parallel-group study. 144 *H. pylori*-infected patients were recruited in the study and randomly assigned by a computer program into three groups: Group SRAC was treated with *Saccharomyces boulardii* powder + triple therapy (S. boulardii powder + rabeprazole + amoxicillin + clarithromycin. After 10 days, only *Saccharomyces boulardii* was given for 14 days). Group BRAC was treated with bismuth quadruple therapy (Rabeprazole + amoxicillin + clarithromycin + bismuth potassium citrate, 10 days). Group RAC was treated with triple therapy (Rabeprazole + amoxicillin + clarithromycin, 10 days). Patients assigned to Group SRAC received rabeprazole (Jichuan Pharmaceutical, Co. Ltd.,

Shanghai, China) 20 mg two times a day (bid); amoxicillin (Zhuhai United Laboratories Co, Ltd, Zhuhai,China) 1.0 g bid; clarithromycin (Abbott Laboratories Ltd, Shanghai, China) 500 mg bid; and *S.* *boulardii* powder (Laboratories Biocodex, Inc., France) 500 mg bid for 10 days. Additionally, *S. boulardii* powder was given for 4 days extend. Patients assigned to Group BRAC received bismuth quadruple therapy for 10 days. Bismuth potassium Citrate (Livzon Pharmaceutical Group, Inc., Zhuhai, China) 220 mg bid, the dosage frequency of other drugs is consistent with Group SRAC. Patients assigned to Group RAC received rabeprazole + amoxicillin + clarithromycin for 10 days, the drug dosage frequency is the same as Group SRAC. Considering the high resistance rate of metronidazole in the Center China population, we took amoxicillin and clarithromycin as our antibiotic choice. For patients who have failed their initial eradication, we provide a quadruple regimen of furazolidone 0.1 g bid + amoxicillin1.0 g bid + S. boulardii powder 500 mg bid + rabeprazole 20 mg bid for 10 days as remedial treatment. Continuing with the administration of S. boulardii powder only for an additional 14 days.

### Study evaluations

Patients were evaluated at three visits: screening (0 day), end of treatment (14 days after the treatment initiation), follow-up the 13C-urea breath test 4 weeks after the therapy completion (44 day). A negative result is judged as *Helicobacter pylori* eradication. The patients were provided with stool for detection at each follow-up. Select patients who have successfully eradicated and provided three times stools for fecal microbiota analysis. After dividing and labeling fecal samples, the researchers immediately placed them in a − 80 ℃ refrigerator for freezing storage. The collected fecal samples were transported via cold chain to Tianjin Nuohe Zhiyuan Co., Ltd. for 16 s rRNA sequencing detection. Short term changes in gut microbiota before and after the eradication of *Helicobacter pylori* with the assistance of *Saccharomyces boulardii* powder combined with triple therapy. Record the patient's symptoms, improvement, medication, adverse reactions (such as nausea, abdominal pain, abdominal distension, diarrhea, and increased symptoms.) and other information in detail. Eradication rates were determined by both ITT- and PP-based analyses. All enrolled patients were included in the ITT analysis, but the PP analysis excluded those patients who dropped out due to side effects, loss to follow-up, or poor compliance.

### 16S rRNA gene amplicon pyrosequencing

According to the manufacturer protocols, we use the Stool DNA Kit (Omega Bio-tek, Norcross, GA, U.S) to extract DNA from the collected fecal samples. To measure the purity and concentration of microbial DNA, we use spectrophotometer and 2% agarose gel electrophoresis. Next step was the V3–V4 region of the bacteria 16S rRNA gene amplicon, inwhere two universal primers 338F(ACTCCTACGGGAGGCAGCAG) and 806R (GGACTACHVGGGTWTCTAAT) was used by PCR. The first step of PCR was 94 °C for 5 min, then did 28 cycles of 94 °C for 30 s, 55 °C for 30 s and 72 °C for 60 s, the final step was 72 °C for 7 min. Each PCR reaction system was consisted of 2X Taq Plus Master Mix (12.5 μL), BSA(3 μL with 2 ng/ul desity), 1.0 μL of each primer (5 μM), ddH_2_O and 30 ng of template DNA in a 25 μl volume. PCR products were collected and purified with QIAquick Gel Extraction Kit (QIAGEN, Germany).

Pooled purified amplicons at equimolar ratios, then paired-end sequenced (2 × 250) on an Illumina HiSeq2500 platform. The raw data were stored in the NCBI Sequence Read Archive (SRA) database. Using Trimmomatic to demultiplex and qualityfilter the raw fast files, and using silva to cluster Operational Taxonomic Units (OTUs) with 97% similarity cutoff. Then UCHIME was used to identify and remove chimeric sequences. The silva (SSU115) 16S rRNA database was analysed by RDP Classifier (http://rdp.cme.msu.edu/) with a confidence threshold of 70%. At each taxonomical level (Phylum, Class, Order, Family, and Genus), one analysis was performed respectively. The diversity of alpha (within samples) and beta (among samples) were analyzed by using Inhouse Perl scripts. The α diversity index was calculated by using Mothur software (version 1.31.2). The R (v3.1.1) software package is used for clustering analysis. UniFrac algorithm uses the information of system evolution to compare the differences of bacteria groups among samples, and makes further statistical analysis of the results^12^.

### Statistical analysis

Data collected from all three groups were represented as the mean ± standard error of the mean (SEM). More than two groups’ datasets were evaulated by one-way ANOVA followed by Newman–Keuls post hoc tests. Among all three groups, statistical significance of nonparametric variables’differences was assessed by the nonparametric Turkey test followed by the Mann–Whitney U test when *P* < 0.05. The statistical analysis was performed by SPSS 25.0 software (Chicago, IL, USA).

## Results

### Baseline characteristics and eradication rates of the study population

A total of 144 individuals with *H. pylori*-positive non-ulcerative dyspepsia were enrolled in this study. These participants were randomly allocated to three treatment groups: SRAC (n = 48), BRAC (n = 48), and RAC (n = 48). Clinical parameters including age, gender, smoking status, BMI, endoscopic diagnosis, history of antibiotic exposure, past medical history and adverse event were consistent across all three groups. Eradication rates, assessed through both ITT and PP analyses, revealed a 75% eradication rate for group SRAC in both analyses. In group BRAC, ITT and PP analyses demonstrated eradication rates of 85.42% and 93.18%, respectively, while group RAC displayed rates of 62.5% (ITT) and 65.2% (PP). Two-group comparison tests established significant disparities in eradication rates across the groups (ITT analysis: SRAC vs. BRAC, *p* = 0.012; BRAC vs. RAC, *p* = 0.042) (PP analysis: SRAC vs. BRAC, *p* = 0.009; BRAC vs. RAC, *p* = 0.021). Particularly, group BRAC exhibited a marginally higher eradication rate compared to the other groups. Common adverse events included nausea, abdominal pain, abdominal distension, and diarrhea. The overall incidence of adverse events differed significantly among the three groups (*p* = 0.018), with a lower frequency of diarrhea observed in group SRAC compared to group BRAC (*p* = 0.01). Remarkably, patients receiving *S. boulardii* treatment exhibited an overall reduction in initial dyspepsia symptoms by the end of the treatment period (Table 1).

**Table 1:** Baseline characteristics, adverse events and *helicobacter pylori* eradication rates among the three treatment groups.

	SRAC (n = 48)	BRAC (n = 48)	RAC (n = 48)	*p* value
Age, yr	50.20 ± 10.97	49.6 ± 10.65	48.77 ± 10.05	0.861
Sex, male/female	23/25	22/26	22/26	0.872
Smoking habits	10	5	9	0.24
BMI	21.91 ± 2.67	22.26 ± 3.02	23.10 ± 3.61	0.288
Endoscopic diagnosis				0.817
Chronic gastritis	27	29	25	
Chronic gastritis with erosion	9	11	12	
Atrophic gastritis	7	4	5	
Gastroesophageal reflux	5	4	6	
History of antibiotic exposure				0.797
Clarithromycin Use	6	9	7	
Amoxicillin Use	19	21	16	
Past medical history				0.679
Chronic gastritis	13	10	11	
Family history of gastric cancer	7	6	4	
Adverse event				
Nausea	3	3	2	
Abdominal pain	0	2	3	
Abdominal distension	5	9	5	
Diarrhea	1	8	6	
Total	9/48 (18.7%)	22/48 (45.83%)	16/48 (33.33%)	0.018
ITT analysis	36/48 (75%)	41/48 (85.42%)	30/48 (62.5%)	0.037
PP analysis	36/48 (75%)	41/44 (93.18%)	30/46 (65.2%)	0.006

Values are presented as mean ± SD or number (%).

### Comparison of fecal microbiome composition in *Saccharomyces boulardii* powder combined with triple therapy between treatment groups

**Figure 1:**
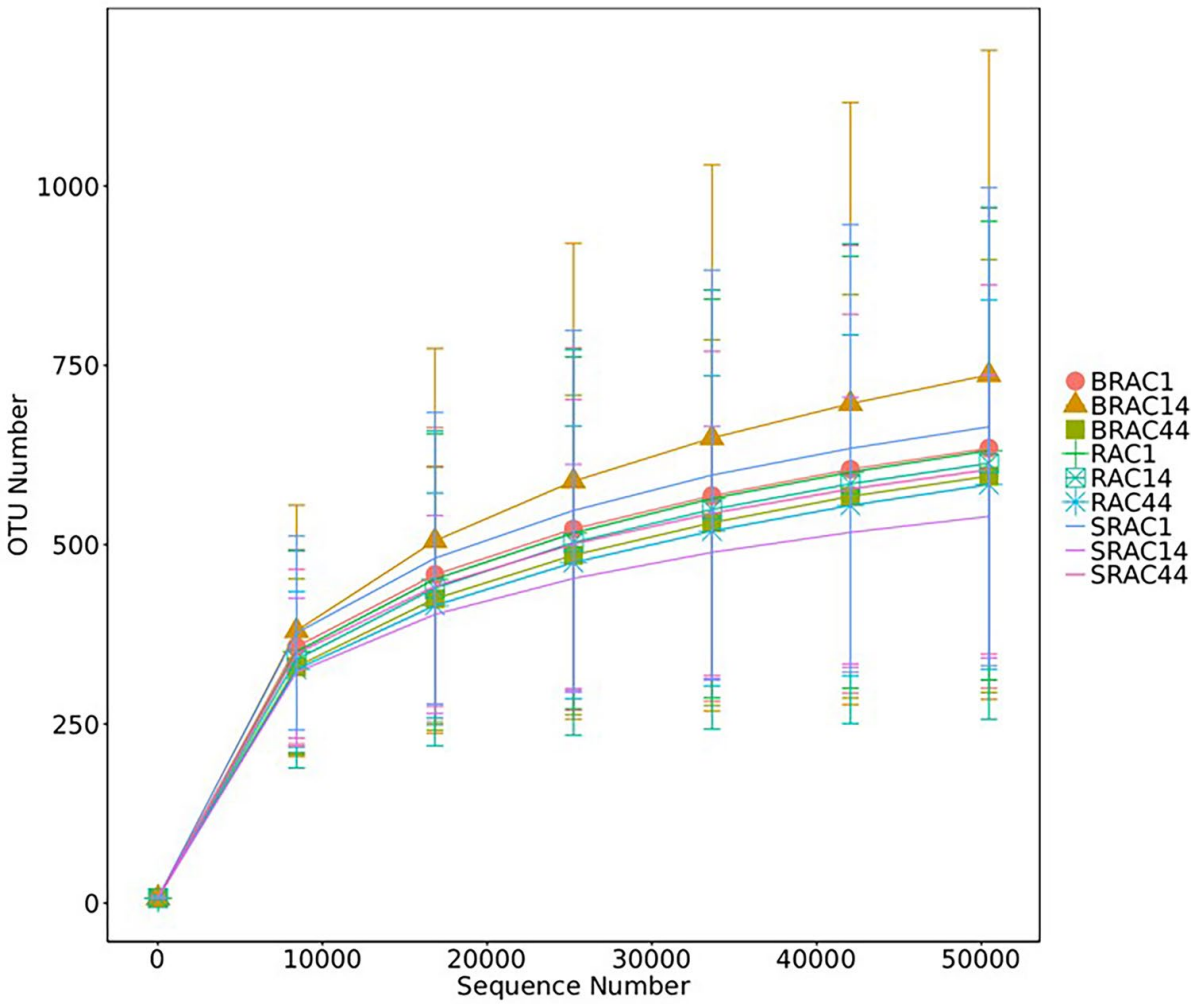
Alpha diversity analysis at the OTU level of all samples’ fecal microbiota.

**Figure 2:**
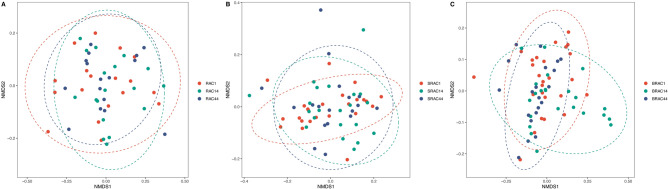
Principal coordinate analysis (PCoA) of microbial communities among the three groups.

**Figure 3:**
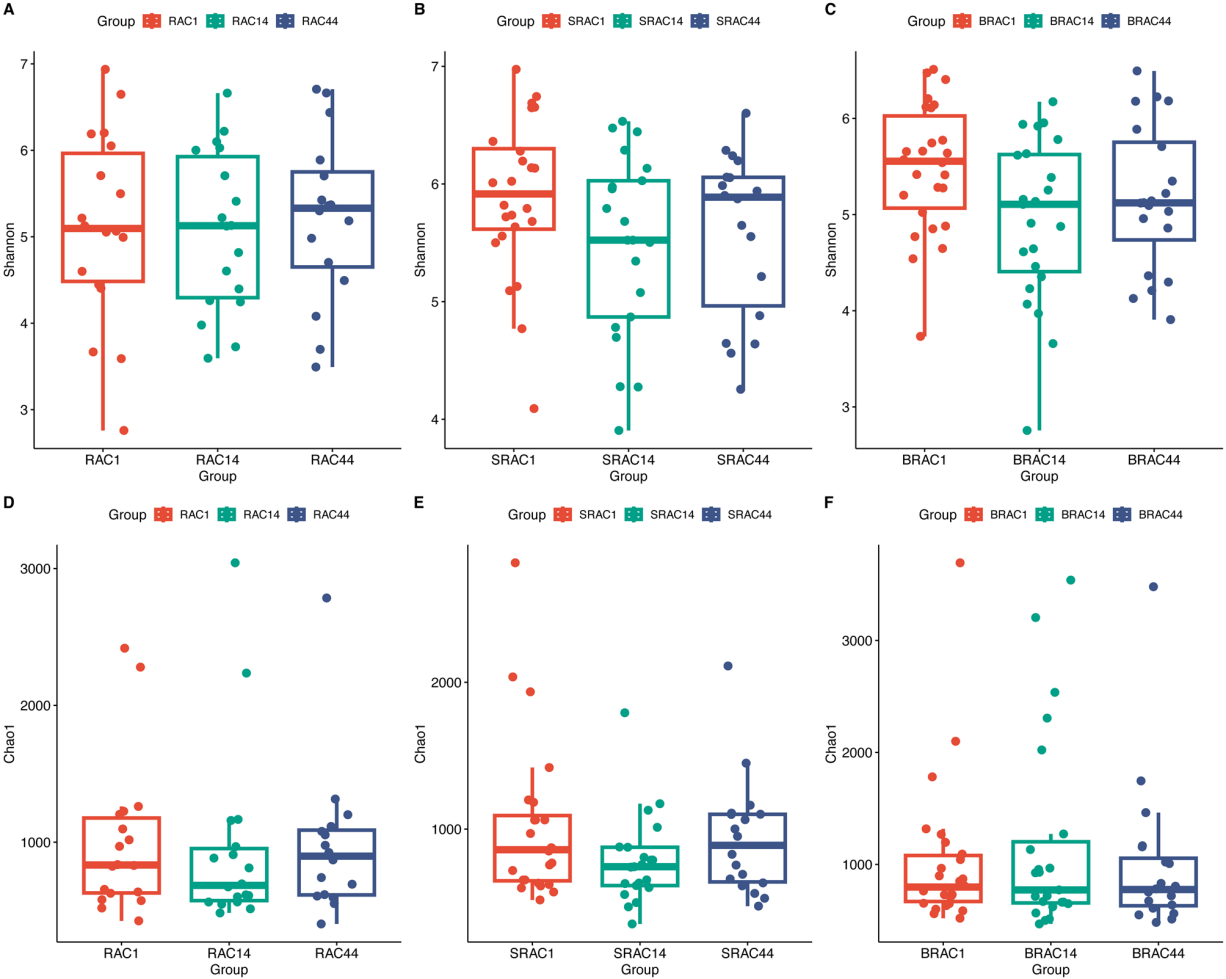
Saccharomyces boulardii modulated the structure and diversity of gut microbiota. (**A**–**C**) Shannon index in different group. (**D**–**F**) Chao1 index in different group. Significant differences only between BRAC group, day0 versus day14 (*p* = 0.022, *p* < 0.05).

For all three groups, fecal samples were subjected to sequencing of variable regions V4–V5 of the 16S rRNA gene using Illumina HiSeq/MiSeq platforms. This approach aimed to assess alterations in the gut microbial community. Operational taxonomic units (OTUs) were utilized to represent results with a 97% homology cutoff value (Fig. 1). Principal coordinates analysis (PCoA) exhibited distinct clustering of microbiota composition for each group (Fig. 2). A comparison of bacterial diversity before and after treatment revealed a significant diversity shift among the BRAC group (Fig. 2B, *p* < 0.05), whereas no significant diversity difference was observed for the SRAC and RAC groups around treatment (Fig. 2A, C). Following 14 days of treatment, the BRAC group displayed a more pronounced alteration in gut microbiota compared to the SRAC and RAC groups (*p* < 0.05), signifying a heightened impact of bismuth quadruple therapy on intestinal microecology. Diversity trends were reflected in Shannon and chao1 index analysis (Fig. 3), with all treatment groups demonstrating reduced diversity on the 14th day, followed by increased diversity on the 44th day. Lower community richness was observed in the RAC group compared to the other two groups (*p* < 0.05).

**Figure 4:**
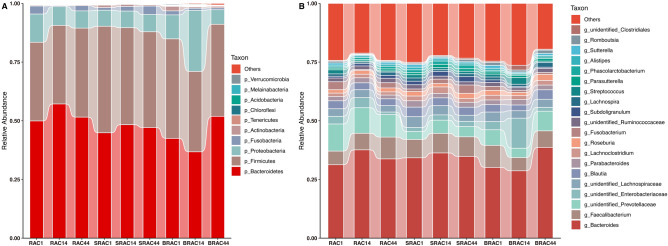
*Saccharomyces boulardii* modulated the composition of gut microbiota at the phylum and genus level. (**a**) Phylum-level taxonomic distributions of the microbial communities in feces. (**b**) Genus-level taxonomic distributions of the microbial communities in feces.

At the phylum level, the relative abundance of dominant taxa identified from sequencing across all three groups is illustrated in Fig. 4A, capturing specific gut microbiota changes. Prior to treatment, no significant variation in phylum levels was observed among the three groups, with *Bacteroidota*, *Firmicutes*, *Proteobacteria*, and *Fusobacteria* accounting for over 95% (*p* < 0.05). In the SRAC group, other prevalent bacteria included *Actinobacteria*, *Acidobacteria*, *Tenericutes*, *Chloroflexi*, *Cyanobacteria*, and *Gemmatimonadetes*. In the BRAC group, other notable bacteria encompassed *Actinobacteria*, *Acidobacteria*, *Chloroflexi*, *Verrucomicrobia*, *Synergistetes*, and *Cyanobacteria*. The RAC group exhibited additional dominant bacteria like *Actinobacteria*, *Chloroflexi*, *Acidobacteria*, *Verrucomicrobia*, *Cyanobacteria*, and *Nitrospirae*. Post-treatment, the relative abundance of *Firmicutes* decreased in the SRAC and BRAC groups, with subsequent recovery trends before treatment by the 44th day. In contrast, the RAC group displayed continuous *Firmicutes* abundance escalation following the 14th and 44th days of treatment. Changes in *Bacteroidota*, *Proteobacteria*, and *Fusobacteria* before and after treatment followed a similar pattern within each group. In the SRAC and RAC groups, relative abundance of *Bacteroidota*, *Proteobacteria*, and *Fusobacteria* declined after 14 days of treatment, rebounding after 44 days and reverting to pre-treatment trends. The converse was observed for the BRAC group, where these bacteria increased post-14-day treatment and decreased after 44 days.

**Figure 5:**
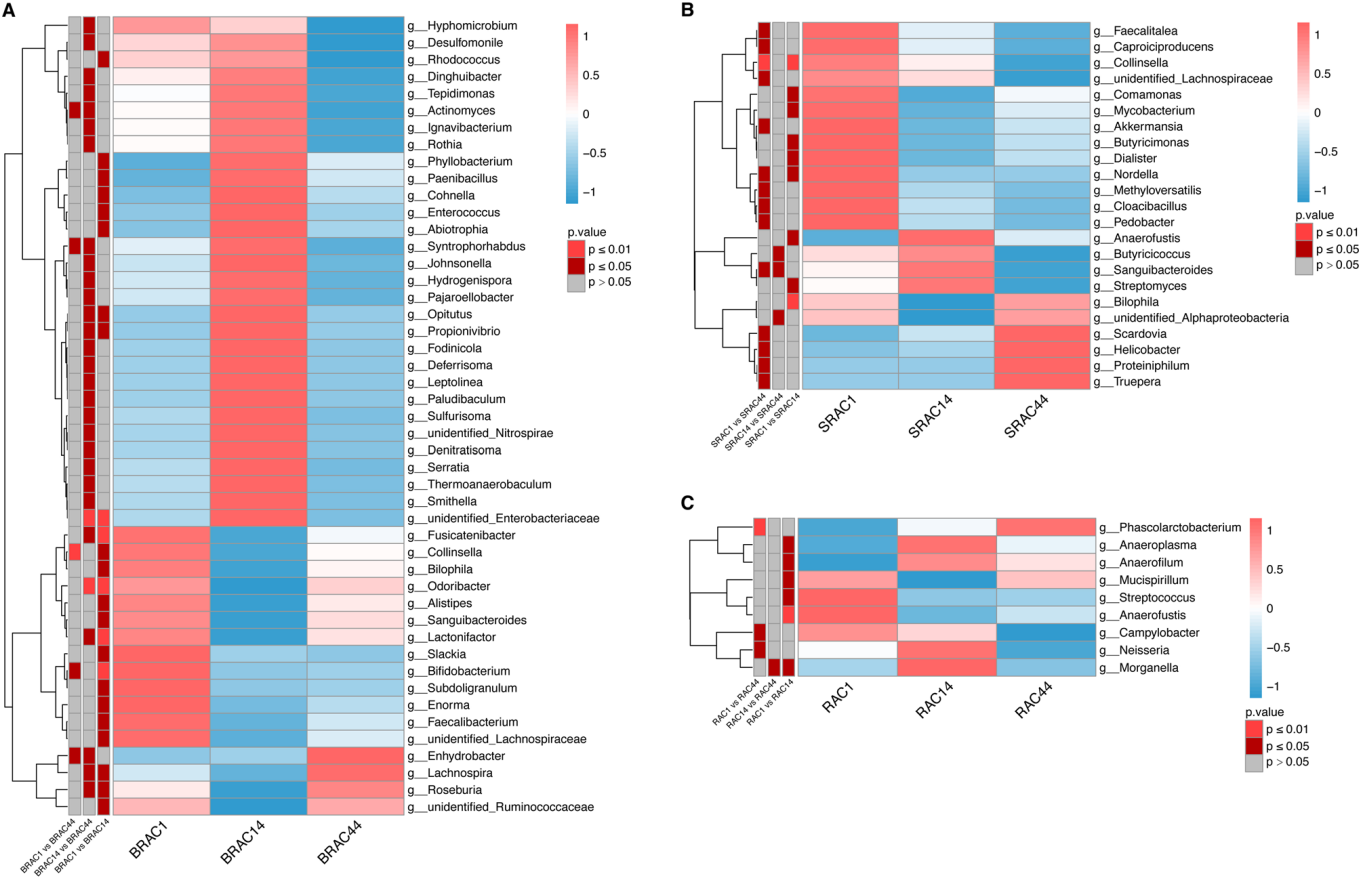
Comparison of species at the genus level with significant difference in abundance among the three groups.

At the genus level, variations in relative bacterial community abundance before and after treatment were evident, marked by distinct changes in bacterial genus composition with notable inter-individual differences. *Bacteroides* emerged as the most prevalent bacterium (Fig. 4B), and further details of intestinal bacteria composition are provided in Fig. 5. The SRAC group featured beneficial bacteria such as *Bacteroides*, *Faecalibacterium*, *Prevotella_9*, *Lachnospiraceae_NK4A136*_group, *Roseburia*, *Lachnospira*, *Lachnoclostridium*, and *Parabacteroides*. After 14 days of treatment, the SRAC group witnessed significant elevation in beneficial bacteria like *Proteiniphilum* and *Anaerofustis *(Fig. 5B, *p* < 0.05), along with a marked decrease in pathogenic bacteria including *Helicobacter*, *Mycobacterium*, *Dialist*, and *Sanguibacteroides* (Fig. 5B, *p* < 0.05). By the 44th treatment day, bacterial community abundance experienced a general increase. In the BRAC group, advantageous bacteria encompassed *Bacteroides*, *Faecalibacterium*, *Prevotella_9*, *Escherichia-Shigella*, *Parabacteroides*, *Lachnoclostridium*, and *Roseburia*. After 14 days of treatment, the BRAC group displayed notable increases in *Faecalibacterium* and *Subdoligranulum* (Fig. 5A, *p* < 0.05). On the 44th treatment day, *Bifidobacterium*, *Collinsella*, and *Actinomyces* showed higher abundances (Fig. 5A, *p* < 0.01), whereas *Roseburia*, *Lachnospira*, and *Fusicatenibacters* decreased significantly compared to pre-treatment levels (Fig. 5A, *p* < 0.05). In the RAC group, advantageous bacteria included *Bacteroides*, *Prevotella_9*, *Fusobacterium*, *Faecalibacterium*, *Escherichia-Shigella*, *Parabacteroides*, *Roseburia* and *Sutterella*. Following 14 days of treatment, the RAC group exhibited an increase in beneficial bacteria like *Phascolarctobacterium* (Fig. 5C, *p* < 0.05), coupled with a decrease in pathogenic bacteria such as *Campylobacter*, *Streptococcus*, *Scardovia*, *Neisseria* and *Morganella* (Fig. 5C, *p* < 0.05). By the 44th day of treatment, bacterial community abundance had risen compared to baseline. In summary, diverse treatment regimens yielded distinct impacts on intestinal microecology, with bismuth quadruple therapy displaying a more pronounced short-term interference on gut microbiota.

## Disscusion

*Helicobacter pylori*, a prevalent Gram-negative microaerobic bacterium, has been a subject of continuous research since Warren and Marshall successfully cultured it from gastric mucosa in 1982. The widely employed standard PPI triple therapy since 1996, involving a proton pump inhibitor along with two antibiotics, has emerged as a prominent anti-*Helicobacter pylori* treatment globally, with treatment durations spanning 7–10 or even 14 days^13–15^. However, in recent years, escalating *Helicobacter pylori* drug resistance has led to the endorsement of bismuth quadruple therapy as the primary treatment approach in regions confronting high clarithromycin resistance, as recommended by the Maastricht and Toronto consensus^16–18^. Although bismuth quadruple therapy boasts a high eradication rate, it is accompanied by a significant incidence of associated side effects, including nephrotoxicity and potentially severe conditions like bismuth encephalopathy^19^. So it is crucial to urgently discover a new type of drug with high safety and effectiveness that can replace bismuth agents for the treatment of *Helicobacter pylori*. The integration of authoritative guidelines on *Helicobacter pylori* resistance also highlights the necessity of adding probiotics^20^.In light of emerging trends, microbiota research has gained prominence, prompting increased scholarly interest in harnessing probiotics as novel therapeutic regimens or supplementary agents for anti-*Helicobacter pylori* treatment^8,21^. In this context, our study endeavors to assess and compare the efficacy and safety of *Saccharomyces boulardii* in eradicating *Helicobacter pylori* combined with standard PPI triple therapy. Furthermore, our investigation committed to observing the changes in gut microbiota during the treatment, in order to find the potential correlations between gut microbiota and clinical efficacy.

Our randomized controlled trial stands as the sole recent experiment comparing the clinical efficacy of *Saccharomyces boulardii* combined with standard PPI triple therapy, simply standard PPI triple therapy, and a 10-day bismuth quadruple therapy. In a recent clinical meta ananlysis involving 15 RCT in children total 2156 patients, it was found that, *Saccharomyces boulardii* in combination with standard triple therapy was more effective than standard triple therapy alone and has a significantly lower incidence of total adverse events^22^. Another meta-analysis included 11 RCT with a total of 2200 patients, it was also mentioned that the addition of *S. boulardii* significantly increased the eradication rate and decreased some therapy-related side effects^23^. The novel and unique aspect of our research lies in exploring whether *Saccharomyces boulardii* can replace bismuth agents or can increase the eradication rate in the treatment of *Helicobacter pylori*. It is a worth noting that the eradication rate of the *Saccharomyces boulardii* combined with standard PPI triple therapy group is much higher than the standard PPI triple therapy group, but still significantly lower than that of the bismuth quadruple group. In a comprehensive clinical study incorporated 1620 participants, rigorously compare the clinical efficacy of bismuth quadruple therapy, standard triple therapy, and concomitant therapy. The results showed that the eradication rates were 90%, 84%, and 86%respectively. Bismuth quadruple therapy is the preferred first-line treatment option^24^. Similarly, for the regimen of omeprazole + clarithromycin + amoxicillin + bismuth potassium citrate, the eradication rate was as high as 93.7%. Even the eradication rate of drug-resistant strains far exceeded the expected 40%, reaching 84.6%^25^. In line with these findings, our study reports a bismuth quadruple therapy eradication rate of 93.94%, basically the same as them. Furthermore, our assessment of adverse reaction occurrences unveiled a substantially lower overall incidence in the *Saccharomyces boulardii* and standard PPI triple therapy group compared to the bismuth quadruple therapy cohort.

Probiotics, as active microorganisms, contribute to intestinal microbiota stability via anti-inflammatory and antioxidant mechanisms, thereby maintaining gut microbiota balance and inhibiting the proliferation of antibiotic resistant gut microbiota^26,27^. A recent meta-analysis found that probiotics as adjunctive drugs are effective in improving *Helicobacter pylori* eradication rates and reducing side effects. Analysis found no difference in efficacy between the use of a single probiotic or the combination use. Ranking the effectiveness of probiotics, it was found that the triple therapy with 10 days of adjunctive treatment using *Saccharomyces boulardii* or *Lactobacillus* was the most effective^28^. Furthmore, for *Helicobacter pylori* positive patients who have failed initial treatment, the natural clearance rate of exogenous administration of *Saccharomyces boulardii* alone is as high as 28%^29^. *Saccharomyces boulardii*, a resilient tropical fungus resistant to antibiotics, retains its potency when administered alongside antibiotics without genetic material transfer, thus ensuring safety. Our study capitalizes on *Saccharomyces boulardii*'s capacity for stable colonization in the host intestinal tract after a two-week regimen^30^.

At present, the most important indicator for evaluating treatment plans is eradication rate, but there are still many other issues worth our attention, including antibiotic resistance and the impact of gastrointestinal microbiota. The use of antibiotics is actually a game process between short-term benefits and long-term risks for human health. Studies have also reported the long-term effects of antibiotics on gut microbiota, but individual differences and even intra individual differences are quite significant in the response to the same antibiotic^31^. In the triple therapy for *H. pylori* eradication, the addition of *S. boulardii* CNCM I-745 to the conventional antibiotic eradication therapy for *H. pylori* reduced the abundance of antimicrobial resistance genes, particularly those genes that confer resistance to lincosamides, tetracyclines, MLS-B, and a few genes in the beta-lactams class^32^.

At the phyla level, the composition and proportion of each sample varies at different phyla levels. *Bacteroidetes* and *Firmicutes* have the highest proportion, while the other top ranked phyla are *Proteobacteria*, *Clostridium*, *Actinobacteria*, *Cyanobacteria* and *Acidobacteria*. However, the result is not consistent with metagenomics. In 2022, Masoud et al. reported on the metagenomic changes in the microbiota before and after the addition of *Saccharomyces boulardii* in *Helicobacter pylori* eradication treatment, with *Bacteroides, Clostridium, Ruinoccoccus, Dorea, Faecalibacterium, Vibrio, Thermotalia, and Lactobacillus* being the dominant phylum^33^. However, there is still controversy over the differences in results due to different testing methods. In our study, Differential post-treatment changes in flora were observed across the three groups. After 14 days of treatment, the relative abundance of *Bacteroidetes* increased while the abundance of *Firmicutes* decreased in the *Saccharomyces boulardii* group and the standard triple group, while the changes in the bismuth quadruple treatment group were opposite. A recent microbiota analysis using lansoprazole, amoxicillin, and clarithromycin for the treatment of *Helicobacter pylori*. Although the drugs and doses are different, but the results are similar to ours. There is no significant difference in bacterial diversity before and after treatment^34^.

At the genus level, changes in the microbial community are more complex, but *Bacteroides* is the most common genus of bacteria both before and after treatment. After treatments, the relative abundance of the opportunistic pathogenic bacteria *Escherichia* and *Shigella* increased, with the bismuth quadruple group showing a more significant increase in abundance, ranking second in the bacterial changes after treatment and exhibiting statistical differences. *Shigella* is only present in humans and gorillas and is one of the main bacteria causing diarrhea^35^. This may explain why the bismuth quadruple group has a higher incidence of diarrhea compared to the other two groups. The abundance of *Bacteroidetes* in the *Saccharomyces boulardii* quadruple therapy group increased significantly after treatment. *Bacteroidetes* is a very important bacterium in the human gut and has a complex relationship with the host. Currently, most studies suggest that the genus *Bacteroides* can exert beneficial effects through mechanisms such as immune regulation, metabolism, and nutrition, and may play a protective role in the occurrence of side effects during the quadruple therapy with *Saccharomyces boulardii*^36,37^. The diversity of gut microbiota in the bismuth quadruple treatment group showed significant changes, indicating that bismuth may have a greater interfering effect on gut microbiota during the treatment process.

Our investigation further underscores the significantly reduced overall adverse reaction incidence and diarrhea rates in the *Saccharomyces boulardii* quadruple therapy group in comparison to the standard PPI triple therapy and bismuth quadruple therapy groups. Furthermore, the *Saccharomyces boulardii* and standard triple therapy group demonstrated superior clinical symptom relief and patient tolerance. Notably, the discernible distinctions in gut microbiota structure and function between the probiotic group and other cohorts highlight the regulatory impact of probiotic adjunct therapy on gut microbiota^38^. Consequently, the combined administration of *Saccharomyces boulardii* and standard triple therapy exhibits potential in minimizing post-treatment intestinal flora disturbances. Due to study duration limitations, the combination of *Saccharomyces boulardii* with standard PPI triple therapy fails to enhance the eradication rate achieved by standard PPI triple therapy, nor does *Saccharomyces boulardii* prove a viable replacement for bismuth in clinical treatment. Within the confines of the current sample size, we cautiously refrain from endorsing *Saccharomyces boulardii* as a complete substitute for bismuth or an unequivocal adjuvant in *Helicobacter pylori* treatment. Larger-scale clinical investigations are poised to provide more precise determinations in this regard. In addition, our analysis does not encompass the long-term effects on gut microbiota. Furthemore, for individual patients who failed eradication in each group, we have all provided remedial treatment. Unfortunately, there are still 6 patients who have failed. We all recommend them culturing with susceptibility testing or molecular determination of genotype resistance. There is no specific bacterial genera were found to potentially serve as predictive indicators for evaluating or predicting the eradication effect of *Helicobacter pylori*. In the future, future large-scale, high-quality, and multicenter RCTs need to carrry out to explor the potential microbial impact of *Saccharomyces boulardii* on *Helicobacter pylori* eradication.

## Data Availability

Data will be made available through publication and SRA database (PRJNA992363).
